# The Association between Three Cyclooxygenase-2 Polymorphisms and Hepatocellular Carcinoma Risk: A Meta-Analysis

**DOI:** 10.1371/journal.pone.0118251

**Published:** 2015-03-02

**Authors:** Zhigang Chen, Jiye Zhu, Chaoyuan Huang, Fang Lian, Guobin Wu, Yinnong Zhao

**Affiliations:** 1 Hepatobiliary Surgery Department, Tumor Hospital of Guangxi Medical University, Nanning, P.R.China; 2 Physiology Department, Guangxi Medical University, Nanning, P.R.China; University of Modena & Reggio Emilia, ITALY

## Abstract

**Background:**

A quantity of case-control studies have been performed to address the association between three cyclooxygenase-2(COX-2) polymorphisms (-1195G/A, -765G/C and +8473T/C) and the risk of hepatocellular carcinoma (HCC). However, previous research results are inconsistent. We conducted this meta-analysis to clarify the correlation between these COX-2 polymorphisms and HCC risk.

**Methods:**

The authors searched in PubMed, EMBASE, Google Scholar, CNKI and WanFang database for relevant articles up to April 28, 2014. The data were extracted by two independent reviewers. Odds ratios (ORs) and 95% confidence intervals were calculated.

**Results:**

A total of 8 studies consisting of 2182 cases and 3324 controls were included in this meta-analysis. For COX-2 polymorphism -1195G/A, an association with increased risk was observed under the heterogeneous, homozygous, dominant model. However, COX-2 polymorphisms (-765G/C and +8473T/C) were not related to HCC risk in this study. We also found a similar result in the subgroup analysis of Chinese population that -1195G/A polymorphism, instead of -765G/C or +8473T/C polymorphism, was correlated with the risk of HCC.

**Conclusions:**

Polymorphism -1195G/A of COX-2 might be associated with susceptibility to HCC, but no similar correlations were observed between polymorphisms (-765G/C and +8473T/C) and HCC risk. Further large and well-designed studies are required to validate this association.

## Introduction

Hepatocellular carcinoma (HCC) is the sixth most prevalent cancer and the third cause of cancer-related death in the world, which continues to be a significant public health problem [1]. The development of HCC is a multifactorial and multistep process. A number of risk factors, such as chronic infection of hepatitis B virus or hepatitis C virus, alcoholic intemperance and food aflatoxin contamination, are reported to be involved in carcinogenesis of HCC and are considered as the major contributors to the development of HCC [2, 3]. However, not everyone with those risk factors tends to end with HCC, which implies that there is an inherited element to HCC.

The cyclooxygenase-2 (COX-2) is one of the key enzymes of prostaglandin pathway, since it can convert the arachidonic acid to prostaglandins. Increasing evidence points to COX-2, which contributes to immune evasion, angiogenesis regulation and apoptosis inhibition, as a risk factor in carcinogenesis of HCC [4, 5]. Some studies have revealed COX-2 is overexpressed in many malignant tumors such as HCC and the selective COX-2 inhibitor markedly inhibited the growth of HCC cell *in vitro* and *in vivo* [6–8]. Mechanistically speaking, polymorphisms in the promoter of COX-2 are capable of influencing the COX-2 expression by altering the binding ability of some nucleoproteins enabling a change in the activity of gene transcription [9]. Moreover, large quantities of studies have confirmed the hypothesis that several COX-2 Single-Nucleotide Polymorphisms (SNPs) involving -1195G/A, -765G/C, and +8473T/C (SNP ID: rs689466, rs20417 and rs5275, respectively), were potentially correlated with HCC risk [10–18], but the results were rather controversial and unconvincing. For COX-2-1195G/A, a previous meta-analysis demonstrated that it may contribute to carcinogenesis of HCC, while the study merely included 5 available studies, which is required larger sample size to make its results more persuasive. Therefore, in order to enhance the credibility of the correlation between COX-2-1195G/A and the risk of HCC, we added two more studies containing 195 cases and 255 controls to this meta-analysis and additionally measured it under recessive model. With respect to COX-2-765G/C, it was proved to be associated with HCC risk in three studies [11, 15, 18], but not in other two studies [16, 17]. As to COX-2+8473T/C, although three studies did not attribute HCC to it, a single study tends not to be convincing enough due to small sample size [19]. Hence, we performed a meta-analysis to validate the associations between COX-2-1195G/A, -765G/C and +8473T/C polymorphisms and HCC risk, aiming to acquire better clinical instructions.

## Methods

### Search strategy

We performed a systemic search in PubMed, EMBASE and Google Scholar as well as Chinese databases including China National Knowledge Infrastructure (CNKI) and WanFang database for all the relevant studies utilizing the following search terms: “cyclooxygenase-2” or “COX-2”, “polymorphism” or “SNPs”, “hepatocellular carcinoma” or “liver cancer” (the latest research was updated to April 28, 2014). Additionally, articles in the reference list were manually searched for potentially relevant studies. Publication language was not restricted. Search and literature retrieval were completed independently by two of the authors (Zhigang Chen and Jiye Zhu). Disagreements of the search result were settled by discussion among all the authors.

### Inclusion and exclusion criteria

Studies were included in the meta-analysis if they met the following criteria: (1) the study assessed the association between hepatocellular carcinoma risk and COX-2 polymorphisms (-1195G/A, -765G/C and +8473T/C) (2) the diagnosis of HCC was validated histologically or pathologically. (3) case-control studies (4) data in the studies were adequate to calculate odds ratio (OR) and 95% confidence interval (95%CI). In the condition of multiple studies based on the same or overlapping population from the same research team, the study providing largest population was included. The main criteria for exclusion of publications: (1) family-based or sibling-based association studies (2) the study without control group. (3) duplicated publication (4) master theses and doctor dissertations (5) literature with insufficient data for evaluating OR and 95%CI.

### Data extraction

Two authors (Zhigang Chen and Jiye Zhu) independently extracted data conforming to the inclusion and exclusion criteria from the eligible articles. Any discrepancies in the process were resolved by consensus. Certain data were retrieved from the included studies: the first author’s name, publication time, ethnicity of the research population, the method of detecting genotype, the number of cases and controls, the genotyping distribution of cases and controls, polymorphism site and the Hardy-Weinberg equilibrium (HWE) results.

### Statistical analysis

We measure the strength of the association between COX-2 SNPs (-1195G/A,-765G/C and +8473T/C) and HCC risk by pooled OR with its corresponding 95% CI. The pooled ORs were estimated for allelic comparison (-1195G/A: A vs G, -765G/C: C vs G, +8473T/C: C vs T), homozygote comparison(-1195G/A: AA vs GG, -765G/C: CC vs GG, +8473T/C: data were insufficient for this model) and heterozygote comparison(-1195G/A: GA vs GG, -765G/C: GC vs GG, +8473T/C: TC vs TT), dominant model (-1195G/A: GA+ AA vs GG, -765G/C: GC+ CC vs GG, +8473T/C: TC+ CC vs TT) and recessive model (-1195G/A: GG+GA vs AA, -765G/C: GG+GC vs CC, +8473T/C: TC+ TT vs CC), respectively. The P value of the pooled OR was considered significant if less than 0.05, which was examined by Z test. Heterogeneity across studies was determined by Chi-square test based Q statistic test and I^2^ statistic and the presence of heterogeneity was confirmed if the result was P_Q_ < 0.05 or I^2^≥ 50%. In the condition of existence of heterogeneity, a random-effect model was utilized [20, 21]; otherwise the fixed-effect model was employed to pool the results [22]. Additionally, if data were sufficient, subgroup analyses were conducted by ethnicity. Furthermore, in order to evaluate the stability of results, a sensitivity analysis was conducted. Both Begg’s test and Egger’s test were performed to test whether publication bias existed or not. All the analyses that have been mentioned were completed by STATA v.12.0.

## Results

### Characteristics of included studies

**Fig 1:**
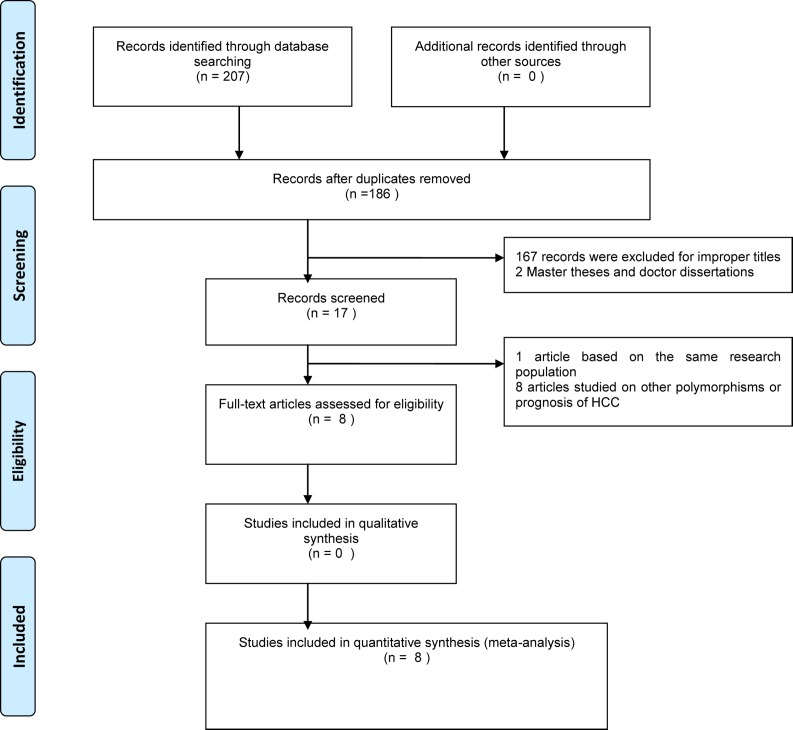
Flow chart of study selection.

As shown in the Fig. 1, 207 articles were found with the search strategy and then 21 duplicates are removed (see S1 Support Information). After reading the titles or the abstracts, 167 records with improper titles, 8 articles studying on other polymorphisms or prognosis of HCC and one article based on the same research population was also excluded. Meanwhile, being a master’s thesis or doctoral dissertation, another 2 publications were discarded [12]. Eventually, 8 studies met the criteria were included in the meta-analysis of which 4 articles were in English and other 4 in Chinese (see S2 Support Information). The characteristics of included articles were showed in Table 1. For -1195G/A polymorphism of COX-2, we included 7 articles including 1882 cases and 2424 controls (for subgroup analysis of Chinese:1558 cases and 2040 controls). 5 articles about -765G/C consisting of 1117 cases and 1997 controls(for subgroup analysis of Chinese:868 cases and 1738 controls) and 3 articles of +8473T/C involving 1207 cases and 1207 controls were included as well. All these studies were case-control study, and two of them merely provided the adjusted OR [14, 18].

**Table 1:** Characteristics of studies included in the meta-analysis.

	First author	Year	Ethnicities	Genotyping method	Number of Cases and controls	Genotype distribution(case/control)			HWE	
−1195G/A						GG	GA	AA	
	Gharib,A^16^		2014	Egyptian	PCR	120/130	17/31	60/66	43/33	0.859
	Mohamed,F^10^		2014	Egyptian	PCR	75/125	12/40	49/22	14/63.	<0.001
	Chang,W,S^17^		2012	Chinese	PCR	298/300	70/74	144/145	84/81	0.570
	Fan,X,J^14^		2011	Chinese	PCR	780/780	204/205	390/381	186/194	0.523
	Akkiz,H^18^		2011	Turkish	PCR	129/129	2/2	36/32	91/95	0.708
	Liu,L,F^13^		2010	Chinese	PCR	210/420	31/101	110/216	69/103	0.557
	Xu,D,K^16^		2008	Chinese	PCR	270/540	52/119	125/287	93/134	0.138
−765G/C							GG	GC	CC	
	Gharib,A^16^		2014	Egyptian	PCR	120/130	86/85	30/39	4/6	0.579
	He,J^15^		2012	Chinese	PCR	300/900	223/772	67/118	10/10	0.027
	Chang,W,S^17^		2012	Chinese	PCR	298/298	262/250	36/48	0/0	0.139
	Akkiz,H^18^		2011	Turkish	PCR	129/129	79/75	46/39	4/15	0.009
	Xu,D,K^16^		2008	Chinese	PCR	270/540	233/515	37/25	0/0	0.582
+8473T/C							TT	TC	CC	
	Chang,W,S^17^		2012	Chinese	PCR	298/298	195/201	103/97	0/0	<0.001
	Fan,X,J^14^		2011	Chinese	PCR	780/780	509/497	235/258	36/25	0.222
	Akkiz,H^18^		2011	Turkish	PCR	129/129	65/58	56/62	8/9	0.161

### Quantitative synthesis

**Fig 2:**
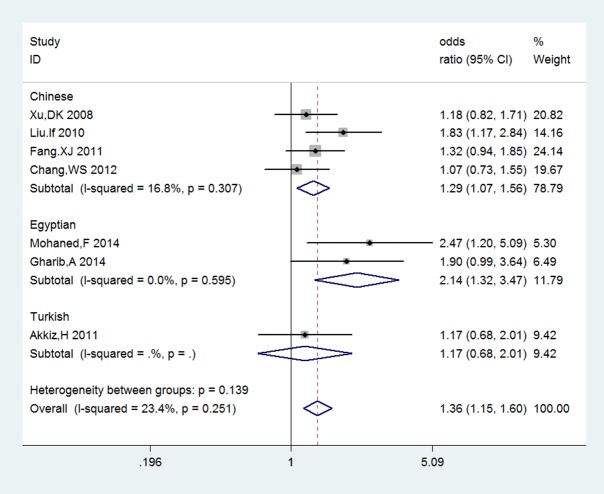
Forest plots of sub-category of ethnicity for COX-2-1195G/A and HCC risk under the dominant genetic model: GA+AA vs GG. The squares and horizontal lines correspond to the study specific odds ratios and 95% confidence intervals. The diamond represents the summary odds ratio and 95% confidence interval.

**Fig 3:**
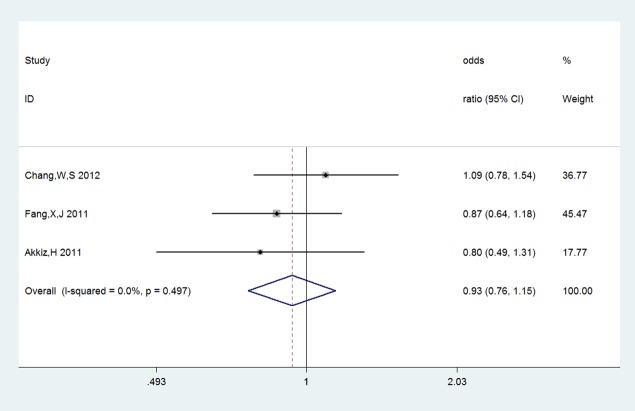
Forest plots of COX-2+8473T/C and HCC risk under the dominant model: TC+CC vs TT. The squares and horizontal lines correspond to the study specific odds ratios and 95% confidence intervals. The diamond represents the summary odds ratio and 95% confidence interval.

**Fig 4:**
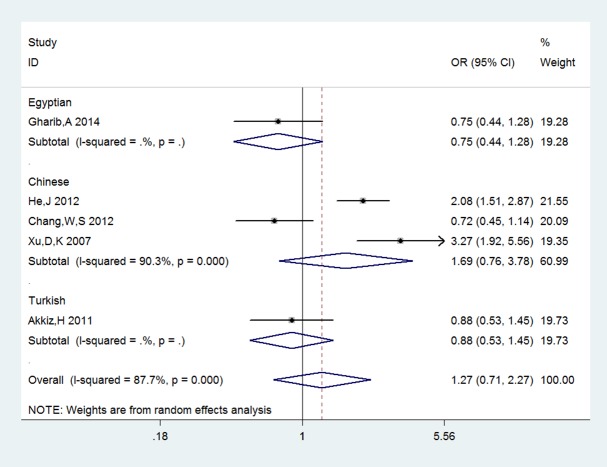
Forest plots of sub-category of ethnicity for COX-2-765G/C and HCC risk under the dominant model: GC+CC vs GG. The squares and horizontal lines correspond to the study specific odds ratios and 95% confidence intervals. The diamond represents the summary odds ratio and 95% confidence interval.

Table 2 showed the main meta-analysis results of relationships between three COX-2 polymorphisms and HCC risk for all population. The results indicated that COX-2-1195G/A exhibited the obvious association with HCC risk in heterogeneous, homozygous comparison and dominant model (GA vs GG: OR = 1.558, 95%CL 1.055–2.303, *P*_*A*_ = 0.026; AA vs GG: OR = 1.466, 95%CL 1.194–1.801, *P*_*A*_ = 0.000; GA+AA vs GG: OR = 1.356, 95%CL 1.148–1.602, *P*_*A*_ = 0.000, **Fig. 2**). However, there was no association in allelic comparison and recessive model (A vs G: OR = 1.118, 95%CL 0.947–1.3190, P_*A*_ = 0.186; GG+GA vs AA: OR = 0.982, 95%CL 0.692–1.392, P_*A*_ = 0.917). Under the dominant model, recessive model as well as other three comparisons, no evidence supported obvious correlations between COX-2+8374T/C and -765G/C and HCC risk (**+8374T/C**: C vs T: OR = 0.986, 95%CL 0.863–1.126 P_*A*_ = 0.833; TC vs TT: OR = 0.927, 95%CL 0.752–1.144, P_*A*_ = 0.480; TC+CC vs TT: OR = 0.934, 95%CL 0.760–1.147, P_*A*_ = 0.513, **Fig. 3**; TC+TT vs CC: OR = 0.764, 95%CL 0.482–1.210, P_*A*_ = 0.252; -**765G/C**: C vs G: OR = 1.223, 95%CL 0.700–2.136, P_A_ = 0.480; GC vs GG: OR = 1.323, 95%CL 0.764–2.290, P_*A*_ = 0.317; CC vs GG:OR = 0.863, 95%CL 0.164–4.530, P_*A*_ = 0.861; GC+CC vs GG: OR = 1.269, 95%CL0.710–2.274, P_*A*_ = 0.423, **Fig. 4**; GG+GC vs CC: OR = 1.195, 95%CL 0.244–5.844, P_*A*_ = 0.826).

**Table 2:** The meta-analysis results of association between Cyclooxygenase-2 polymorphisms and hepatocellular carcinoma risk.

SNPs	Contrast model	OR(95%CL)	P_A_	Test for heterogeneity		publication bias (Egger’s test)	
				I_2_ (%)	*P*	t	*p*
−1195G/A							
	A vs G	1.118(0.947–1.319)	0.186	65.1	0.009	0.12	0.909
	GA vs GG	1.558(1.055–2.303)	0.026	72.5	0.001	1.31	0.247
	AA vs GG	1.466(1.194–1.801)	0.000	31.5	0.187	−0.26	0.808
	GA+AA vs GG	1.356(1.148–1.602)	0.000	23.4	0.251	2.10	0.090
	GG+GA vs AA	0.982(0.692–1.392)	0.917	82.1	0.000	0.71	0.508
+8473T/C							
	C vs T	0.986(0.863–1.126)	0.833	0.0	0.682	0.02	0.989
	TC vs TT	0.927(0.752–1.144)	0.480	0.0	0.471	−0.34	0.790
	TC+CC vs TT	0.934(0.760–1.147)	0.513	0.0	0.497	−0.37	0.775
	TC+TT vs CC	0.764(0.482–1.210)	0.252	0.0	0.374	-	-
−765G/C							
	C vs G	1.223(0.700–2.136)	0.480	41.38	0.000	−0.16	0.882
	GC vs GG	1.323(0.764–2.290)	0.317	85.2	0.000	−0.67	0.549
	CC vs GG	0.863(0.164–4.530)	0.861	13.56	0.001	−1.39	0.398
	GC+CC vs GG	1.269(0.710–2.274)	0.423	87.7	0.000	−0.85	0.460
	GG+GC vs CC	1.195(0.244–5.844)	0.826	12.63	0.002	1.18	0.447

Subgroup analysis by ethnicity (results are also listed in **Table 3**) indicated that -1195G/A polymorphisms of COX-2 was remarkably associated with HCC risk in Chinese population (A vs G: OR = 1.158, 95%CL 0.972–1.381, P_*A*_ = 0.104; GA vs GG: OR = 1.203, 95%CL 0.987–1.467, P_*A*_ = 0.068; AA vs GG: OR = 1.472, 95%CL 1.180–1.838, P_*A*_ = 0.001, GA+AA vs GG:OR = 1.290, 95%CL 1.069–1.555, P_*A*_ = 0.008, GG+GA vs AA: OR = 0.815, 95%CL 0.621–1.068, P_*A*_ = 0.137). It seemed that COX-2-765G/C was not correlated with susceptibility to HCC in Chinese population (C vs G: OR = 1.665, *95%CL* 0.783–3.541, *P*_*A*_ = 0.186; GC vs GG: OR = 1.657, *95%CL* 0.747–3.675, *P*_*A*_ *=* 0.234; GC+CC vs GG: OR = 1.691, *95%CL* 0.757–3.777, *P*_*A*_ *=* 0.200). As only one or two studies of the Turkish or Egyptian were included, the analysis result tended to be less reliable, of which detailed data were not showed in this study.

**Table 3:** The meta-analysis results of association between Cyclooxygenase-2 polymorphisms and hepatocellular carcinoma risk (For Chinese population).

SNPs	Contrast model	OR(95%CL)	P_A_	Test for heterogeneity	
				I_2_ (%)	*P*
−1195G/A					
	A vs G	1.158(0.972–1.381)	0.104	68.0	0.025
	GA vs GG	1.203(0.987–1.467)	0.068	11.3	0.337
	AA vs GG	1.472(1.180–1.838)	0.001	30.2	0.231
	GA+AA vs GG	1.290(1.069–1.555)	0.008	16.8	0.307
	GG+GA vs AA	0.815(0.621–1.068)	0.137	66.6	0.030
−765G/C					
	C vs G	1.665(0.783–3.541)	0.186	90.1	0.000
	GC vs GG	1.657(0.747–3.675)	0.234	89.9	0.000
	GC+CC vs GG	1.691(0.757–3.777)	0.200	90.3	0.000

### Publication bias and sensitivity analysis

The Begg’s test and Egger’s test were applied to assess the publication bias of included literature. The shapes of funnel plot were symmetrical, which have not implied the existence of publication bias. All the Egger’s test results of three polymorphisms were demonstrated in **Table 2**, all *p* values were greater than 0.05 and thus there was no obvious publication bias in the meta-analysis.

Sensitivity analysis was performed to evaluate the stability of the result. Each data set was omitted individually to investigate the impact of a single study on the pooled ORs. The exclusion of any single study did not alter the overall conclusion, indicating that results were reliable.

## Discussion

As a crucial enzyme of prostaglandin pathway, COX-2 attracted more attention in the previous studies. COX-2 was confirmed to be over-expressed in many malignant and metastatic cancers, including HCC [10, 23], which indicated potential correlation may exist. However, results of former studies are conflicting, which implies that final conclusion is still unclear. Thus, a meta-analysis of relationships between three cyclooxygenase-2 polymorphisms and hepatocellular carcinoma risk was conducted in this study.

In our meta-analysis, relationships between three COX-2 polymorphisms have been investigated. For-1195G/A, it has been fully discussed in a previous meta-analysis that aims to clarify the relationship between COX-2-1195G/A and digestive system cancers, which, however, seems not to include HCC [24]. Therefore, we carried out a meta-analysis about the association between COX-2-1195G/A and HCC. We noticed that a correlation exists between COX-2-1195G/A and HCC risk under the homogeneous and dominant model (GA vs GG: OR = 1.558, 95%CL 1.055–2.303, *P*_*A*_ = 0.026; AA vs GG: OR = 1.466, 95%CL 1.194–1.801, *P*_*A*_ = 0.000; GA+AA vs GG: OR = 1.356, 95%CL 1.148–1.602, *P*_*A*_ = 0.000). Besides, it also appeared to be associated with HCC risk in the heterogeneous comparison (GA vs GG: OR = 1.558, 95%CL 1.055–2.303, *P*_*A*_ = 0.026). This result is similar to a previous meta-analysis including merely 5 articles [25], which renders it less persuasive. The credibility of this conclusion has been enhanced after we collected more articles providing larger quantities of cases and controls. To be specific, the statistical significance of homozygous and dominant model in these two studies is the same, but P value of ours is much lower than that of the previous study(homozygous model:P_ours_ = 0.000, P _Bu, X., *et al*._ = 0.001; dominant model: P_ours_ = 0.000, P _Bu, X., *et al*._ = 0.011)[25]. The lower the P value, the more certain it is that COX-2-1195G/A correlates with HCC risk under these two models. Furthermore, in the former study, P value of heterogeneous model is 0.070, compared to 0.026 of our study, which shows a statistical significance [25]. Aiming to complete the research of the association between COX-2-1195G/A and HCC risk, we also measured it under recessive model, which seems to be omitted in other research. Meanwhile, our result of ethnicity subgroup analysis implies that the association is also evident in Chinese population (AA vs GG: OR = 1.472, 95%CL 1.180–1.838, P_*A*_ = 0.001, GA+AA vs GG:OR = 1.290, 95%CL 1.069–1.555, P_*A*_ = 0.008).Thus, we can deduce that COX2-1195G/A tends to facilitate the development of HCC to some extent, but this result was still required further studies to confirm the correlation with HCC risk.

The COX2-765G/C has been reported to be strongly associated with skin, nasopharyngeal, gastric carcinoma [26–28]. In addition, two previous meta-analyses have showed this SNP may be involved in the gastrointestinal tumor risk [29, 30]. More complicated and conflicting results were found when it comes to HCC. Three former studies revealed an association between the COX2-765G/C and HCC risk [11, 15, 18], but a reverse situation was reported in other two studies [16, 17], in which no such correlation exists between COX2-765G/C and the susceptibility to HCC. One of the possibilities accounting for inconsistent results is that sample sizes of publications included in those studies are fairly small which suggests the level of evidence is relatively low. Hence we conducted a meta-analysis to clarify the relationship between COX2-765G/C and HCC risk. In our study, no significant association between them has been observed under any available genetic models. Although we performed the subgroup analysis based on ethnicity to eliminate the heterogeneity among these studies, the heterogeneity still existed in Asian. Similarly, there is no correlation between the COX2-765G/C and HCC risk for Asia population.

The COX-2 +8473T/C is relevant to oral squamous cell carcinoma and breast malignancy and a study showed that the COX-2 +8473T/C polymorphism is not associated with lung cancer risk proved by meta-analysis [31, 32]. Three studies reported the COX-2 +8473T/C are not associated with the susceptibility to HCC [19]. A single case-control study with small sample size might not totally reveal the complex genetic relationship between them. Therefore, we still analyzed the data from three included studies of the COX-2 +8473T/C and the result of meta-analysis suggested that COX-2+8473T/C could not exert any influence on susceptibility to HCC.

Before we made a deduction from a meta-analysis, a number of limitations and shortcomings should be considered. Firstly, No certain studies using a prospective design confirm the correlation between three COX-2 polymorphisms (-1195G/A,-765G/C and +8473T/C) and HCC risk. None of the studies included in our meta-analysis is not retrospective hospital-based case-control study and thus a prospective study should be applied to study the causal relationship between three COX-2 polymorphisms (-1195G/A,-765G/C and +8473T/C) and HCC risk. Secondly, the quantity of studies and cases involved are limited, especially in the analysis of COX-2-765G/C and +8473T/C. To be more specific, only 5 studies about COX-2-765G/C and 3 studies about COX-2+8473T/C were included. The majority of articles in this study were about Chinese and Egyptian and no studies of other races were included in this meta-analysis. The meta-analysis suggested the possible association between COX-2-1195G/A and HCC risk, whilst the correlation in other ethnic groups remain ambiguous as before. Hence large sample size studies targeting to other racial population ought to be designed and conducted in the near future. Finally, some environmental risk factors like HBV/HCV infection, liver cirrhosis and alcohol intake were not considered in this meta-analysis as a result of insufficient data. It is likely that those risk factors may be related to the association between COX-2-1195G/A and HCC risk, but no evidence relevant to the possible interaction was mentioned in the included studies. Apparently, further well-designed studies in which other risk factors are thoroughly considered are essential for casting light on relationships among environmental risk factors, COX-2 polymorphisms and HCC.

In conclusion, the COX-2-1195G/A polymorphism might have an association with HCC risk, but COX-2-765G/C and COX-2+8473T/C are not likely to exert any influence on the susceptibility to HCC. However, data of this meta-analysis were restricted to Chinese, Turkish and Egyptian population and no publications involving other ethnic groups were included. Large samples size and well-designed studies are urgently needed.

## Supporting Information

S1 Checklist PRISMA 2009 Checklist and Meta-analysis on Genetic Association Studies Checklist. (ZIP)

S1 Support Information The full details of the databases searched to identify the studies included in this meta-analysis. (ZIP)

S2 Support Information URLs of articles for reference published in Chinese. (DOCX)
