# Supplementary material for: The Association between Three Cyclooxygenase-2 Polymorphisms and Hepatocellular Carcinoma Risk: A Meta-Analysis
Source: PLoS One. 2015 Mar 2;10(3):e0118251. doi: 10.1371/journal.pone.0118251 (PMC4346398; doi:10.1371/journal.pone.0118251)
Supplement: S2 Support Information — (DOCX) [file pone.0118251.s003.docx]

**Chinese title**: COX-2基因启动子区单核苷酸多态与肝癌遗传易感性的关系

**English title**: Association between single nucleotide polymorphisms in promoter of COX-2 gene and hereditary susceptibility to hepatocellular carcinoma [article in Chinese]

**Reference information**: Xu DK, Zhang XM, Zhao P and Cai JC (2008) Association between single nucleotide polymorphisms in promoter of COX-2 gene and hereditary susceptibility to hepatocellular carcinoma [article in Chinese]. Chin J Hepatobiliary Surg 14: 840-843.

**Web title**: COX-2基因启动子区单核苷酸多态与肝癌遗传易感性的关系 Association between single nucleotide polymorphisms in promoter of COX-2 gene and hereditary susceptibility to hepatocellular carcinoma

**DOI**: 10.3760/cma.j.issn.1007-8118.2008.12.003

**URL**: <http://d.wanfangdata.com.cn/Periodical_zhgdwk200812003.aspx>

**Chinese title**: 环氧化酶2基因-1195G/A基因型和病毒性乙型肝炎相关性肝癌发病风险相关的病例对照研究

**English title**: The relationship between cyclooxygenase-2 gene-1195 G/A genotype and risk of HBV-induced HCC: a case–control study in Han Chinese people [article in Chinese].

**Reference information**: Liu LF, Zhang JL and Lin JS (2010) The relationship between cyclooxygenase-2 gene-1195 G/A genotype and risk of HBV-induced HCC: a case–control study in Han Chinese people [article in Chinese]. Chin JGastroenterol Hepatol 19: 333-335.

**Web title**: 环氧化酶2基因-1195G/A基因型和病毒性乙型肝炎相关性肝癌发病风险相关的病例对照研究 The relationship between Cyciooxygenase-2 gene-1195G/A genotype and risk of HBV-induced HCC: A case-control study in Han Chinese people

**DOI**: [10.3969/j.issn.1006-5709.2010.04.012](http://dx.doi.org/10.3969%2fj.issn.1006-5709.2010.04.012)

**URL**: <http://d.wanfangdata.com.cn/Periodical_wcbxhgbxzz201004012.aspx>

**Chinese title**: 环氧化酶-2765G/C基因多态性对原发性肝癌易感性的研究

**English title**: Cyclooxygenase-2 Polymorphisms and Susceptibility to Hepatocellular Carcinoma [article in Chinese]

**Reference information**: 18. Song X, Cheng SH and Liu C (2011) Cyclooxygenase-2 Polymorphisms and Susceptibility to Hepatocellular Carcinoma [article in Chinese]. The Practical Journal of Cancer: 255-258.

**Web title**: 环氧化酶-2765G/C基因多态性对原发性肝癌易感性的研究 - 中国学术期刊网络出版总库

**DOI**: 10.3969/j.issn.1001-5930.2011.03.009

**URL**: <http://www.cnki.net/KCMS/detail/detail.aspx?QueryID=1&CurRec=1&recid=&filename=SYAZ201103009&dbname=CJFD1112&dbcode=CJFQ&pr=&urlid=&yx=&uid=WEEvREcwSlJHSldSdnQ1ZXI4NjBQL3VFZklvVVBUZ2UvMzlOckptSTRXRW80VzhRQzlhTE9jL1VaN2ZsTG8wPQ==&v=MDQ3NDVMRzRIOURNckk5RmJZUjhlWDFMdXhZUzdEaDFUM3FUcldNMUZyQ1VSTDZmWWVabUZpRG1WYjdMTmpUS2Q>

Or

<http://d.wanfangdata.com.cn/Periodical_syazzz201103009.aspx>

**Chinese title**: COX-2基因单核苷酸多态性与肝细胞癌关联的研究

**English title**: Association of COX-2 gene SNPs with the risk of hepatocellular carcinoma [article in Chinese]

**Reference information**: 22.Fan XJ, Qiu XQ, Yu HP and Zeng XY (2011) Association of COX-2 gene SNPs with the risk of hepatocellular carcinoma [article in Chinese]. Chin J Cancer Prev Treat 18: 405-409.

**Web title**: COX-2基因单核苷酸多态性与肝细胞癌关联的研究 Association of COX-2 gene SNPs with the risk of hepatocellular carcinoma

**URL**: <http://d.wanfangdata.com.cn/Periodical_qlzlzz201106002.aspx>
